# Supplementary material for: Smad7 Sustains Stat3 Expression and Signaling in Colon Cancer Cells
Source: Cancers (Basel). 2022 Oct 12;14(20):4993. doi: 10.3390/cancers14204993 (PMC9599800; doi:10.3390/cancers14204993)
Supplement: Supplementary file 1 [file cancers-14-04993-s001.zip › cancers-1857357-supplementary.pdf]

Supplementary Materials: Smad7 Sustains Stat3 Expression and Signaling in Colon Cancer Cells

Claudia Maresca, Giulia Di Maggio, Carmine Stolfi, Federica Laudisi, Marco Colella, Teresa Pacifico, Antonio Di Grazia, Davide Di Fusco, Daniele Congiu, Andrea Martina Guida, Giuseppe Sica, Ivan Monteleone and Giovanni Monteleone

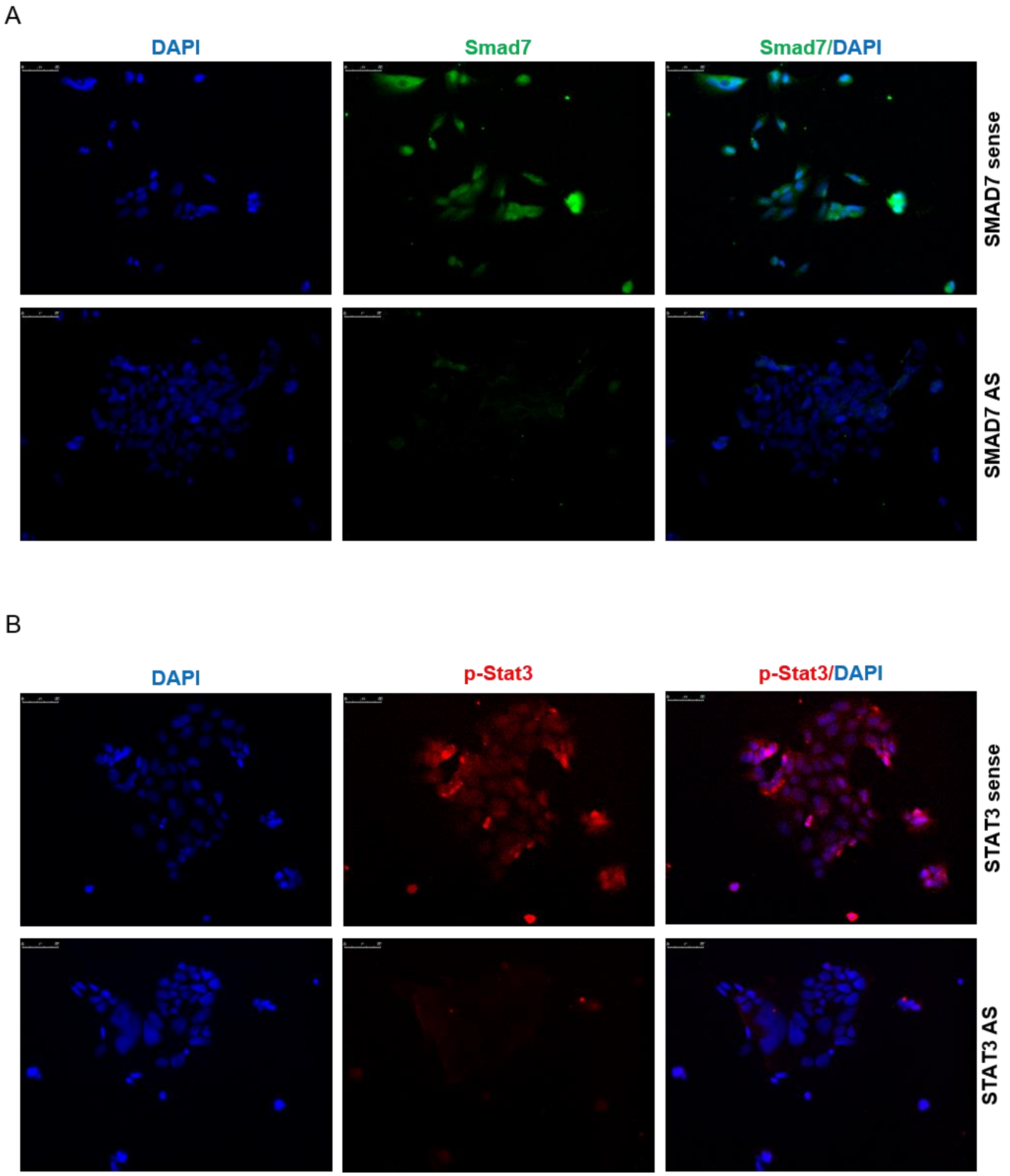

Figure S1. (A) Representative images of single and double-immunofluorescence staining of DLD1 cells transfected with either Smad7 sense or antisense (AS) oligonucleotides as indicated in materials and methods and then analyzed for the expression of Smad7 (green), and DAPI (blue). The scale bars are 50 µm. The figure is representative of three separate experiments in which similar results were obtained. (B) Representative images of single and dou-ble-immunofluorescence staining of DLD1 cells transfected with either Stat3 AS or sense as in-dicated in materials and methods and then analyzed for the expression of p-Stat3 (red), and DAPI (blue). The scale bars are 50 µm. The figure is representative of three separate experiments in which similar results were obtained.

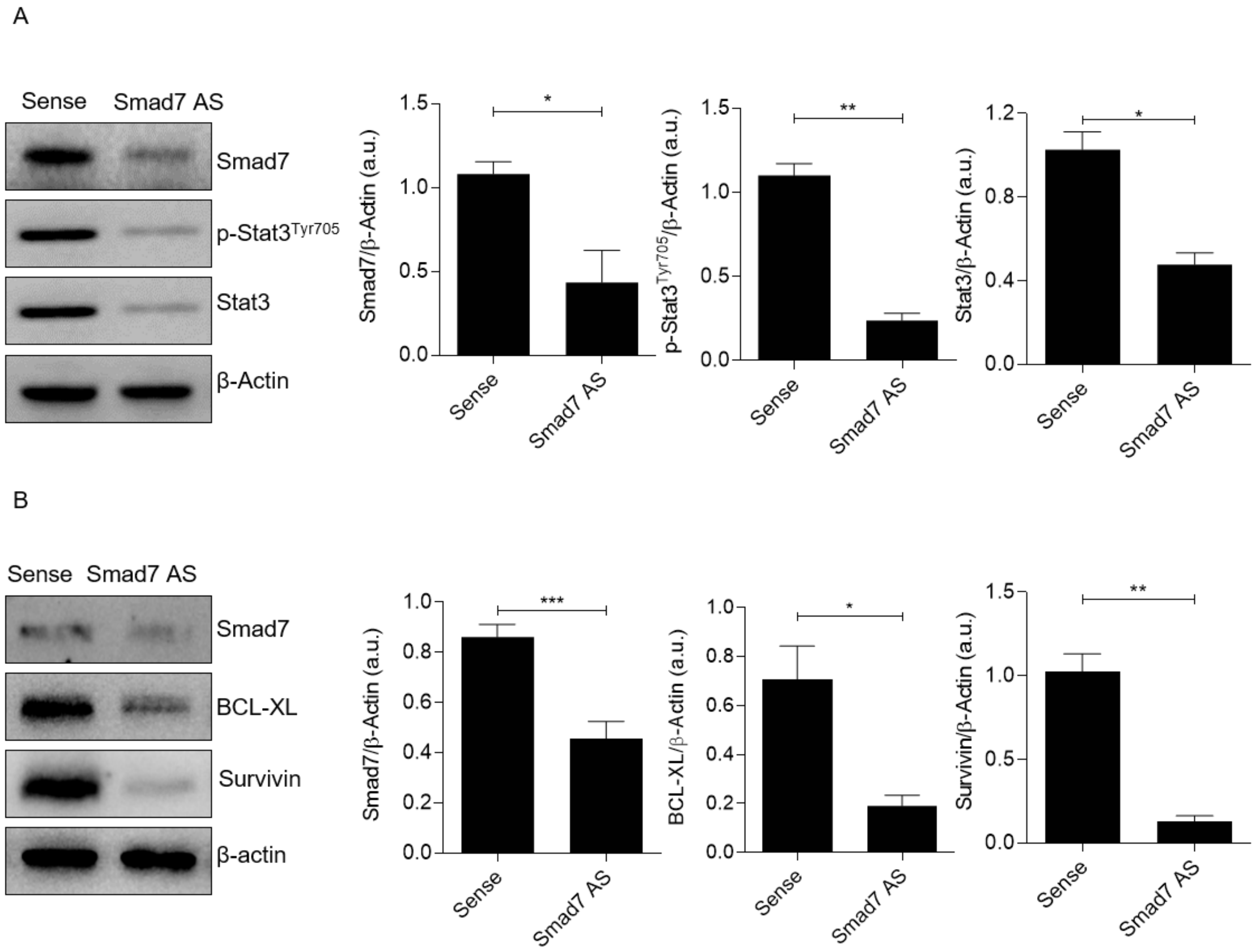

Figure S2. (A) Smad7 antisense oligonucleotide (AS) downregulates Smad7, p-Stat3 Y705 and Stat3 expression in HCT116 cells. Cells were transfected with either Smad7 sense oligonucleotide (sense) or AS (both used at 2 μg/ml) for 24 hours. Smad7, p-STAT3 Y705, total Stat3 and β-actin were analyzed by Western blotting. Representative Western blots are shown. Densitometry analysis of Western blots of all experiments are shown in the right panels. Protein levels are expressed as arbitrary units and data indicate mean ± standard deviation; * $P=0.04$; ** $P<0.0001$. (B) Smad7 AS downregulates protein expression of BCL-xL and survivin in HCT-116. Cells were cultured as above and Smad7, BCL-xL, survivin and β-actin were analyzed by Western blotting. Right panel shows the quantitative analysis of Smad7, BCL-xL, survivin and β-actin as evaluated by densitometry scanning of Western blots. Values indicate the mean ±SEM and differences were evaluated by the two tailed Student's t-test (*$P < 0.05$).

Figure S3

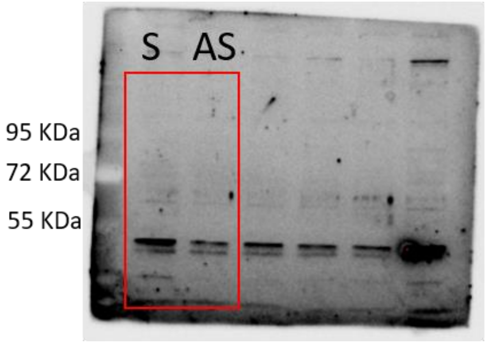

Smad7

S: 703.534,00
AS: 198.403,00

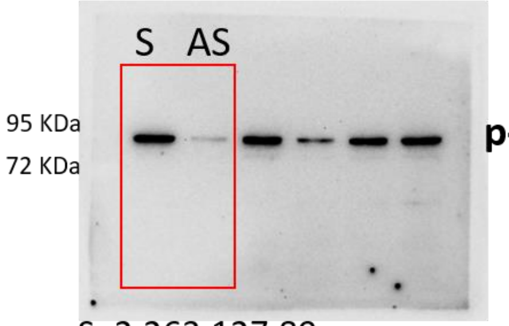

p-Stat3

S: 2.263.127,89
AS: 127.940,89

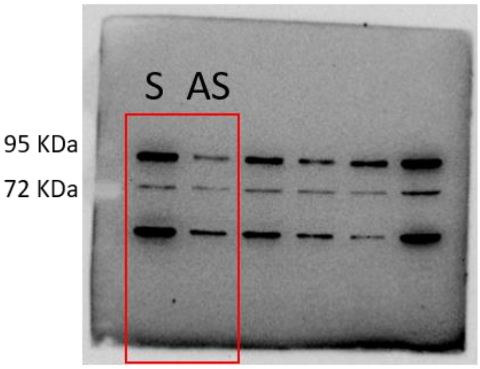

Stat3

S: 1.038.584,00
AS: 108.900,00

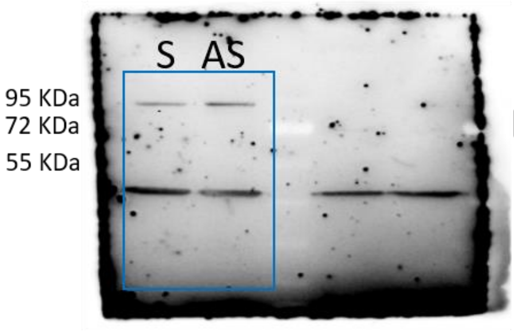

p-Stat1*

S: 715.545,23
AS: 1.122.621,90

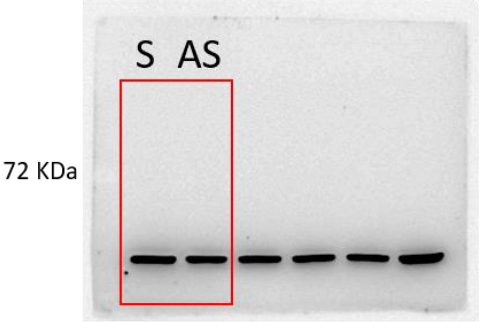

β-actin

S: 6.783.277,32
AS: 5.155.896,49

* This antibody was incubated in a different membrane

Figure S4

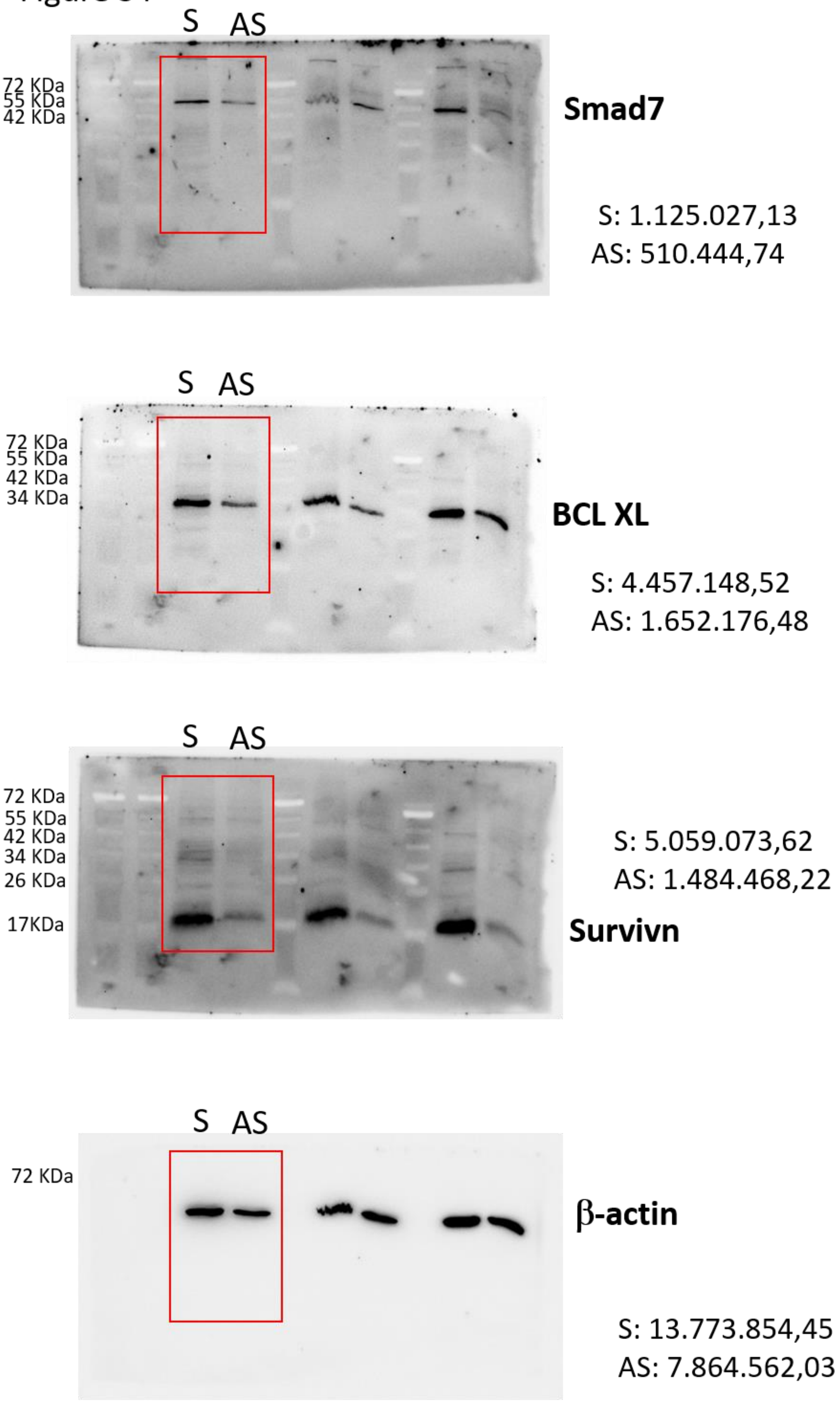
S AS
72 KDa
55 KDa
42 KDa
Smad7
S: 1.125.027,13
AS: 510.444,74
S AS
72 KDa
55 KDa
42 KDa
34 KDa
BCL XL
S: 4.457.148,52
AS: 1.652.176,48
S AS
72 KDa
55 KDa
42 KDa
34 KDa
26 KDa
17KDa
S: 5.059.073,62
AS: 1.484.468,22
Survivn
S AS
72 KDa
β-actin
S: 13.773.854,45
AS: 7.864.562,03

Figure S5

A

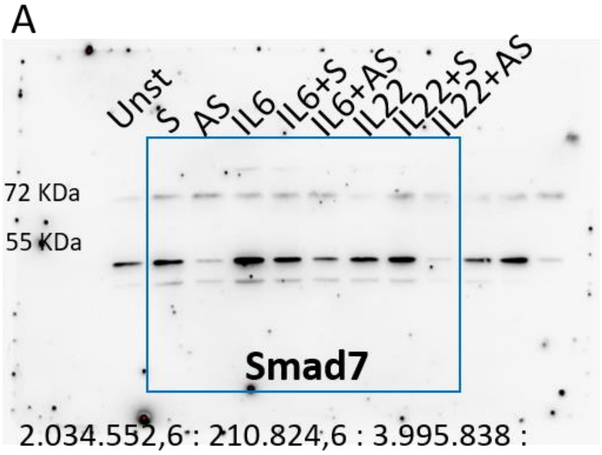

2.034.552,6 : 210.824,6 : 3.995.838 :
1.940.919,2 :737.273,8 : 1.645.657,1 :
2.412.487,3 : 171.077,5

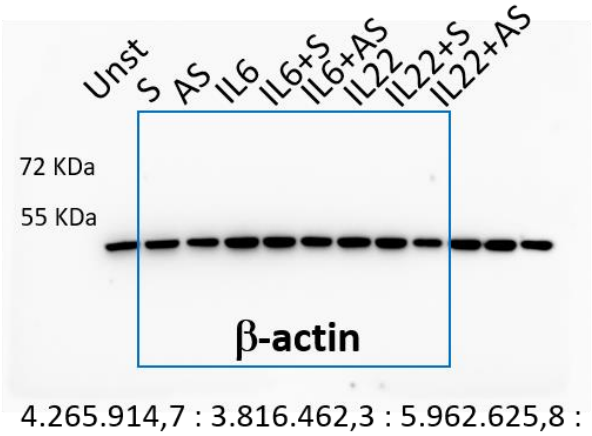

4.265.914,7 : 3.816.462,3 : 5.962.625,8 :
5.088.487,7 :3.890.600,9 : 4.843.015,2 :
4.874.265 : 2.458.271,5

B

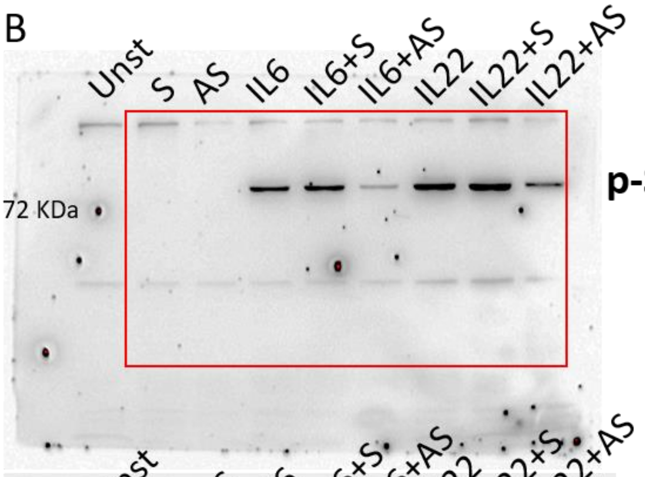

p-Stat3

0 : 0 :
3.916.370,33 : 4.775.196,76 :
848.360,95 : 7.471.642,24 :
8.695.499,67 : 2.379.080,81

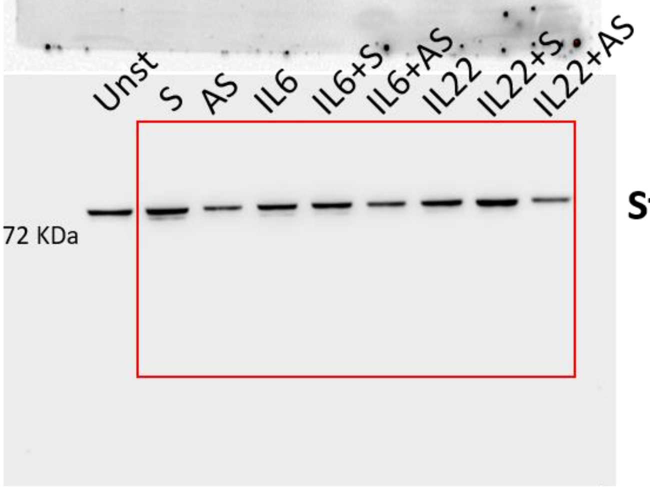

Stat3

10.110.564,80 : 4.234.821,40 :
6.890.864,00 : 7.223.324,00 :
3.903.996,00 : 7.831.507,20 :
9.961.480,40 : 3.821.144,40

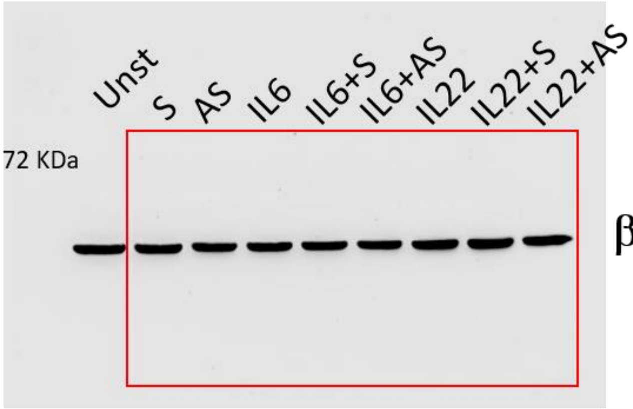

β-actin

17.633.681,57 : 16.785.217,31 :
17.058.589,26 : 16.788.576,47 :
16.993.543,91 : 20.501.221,07 :
19.585.820,95 : 21.317.371,28

Figure S6

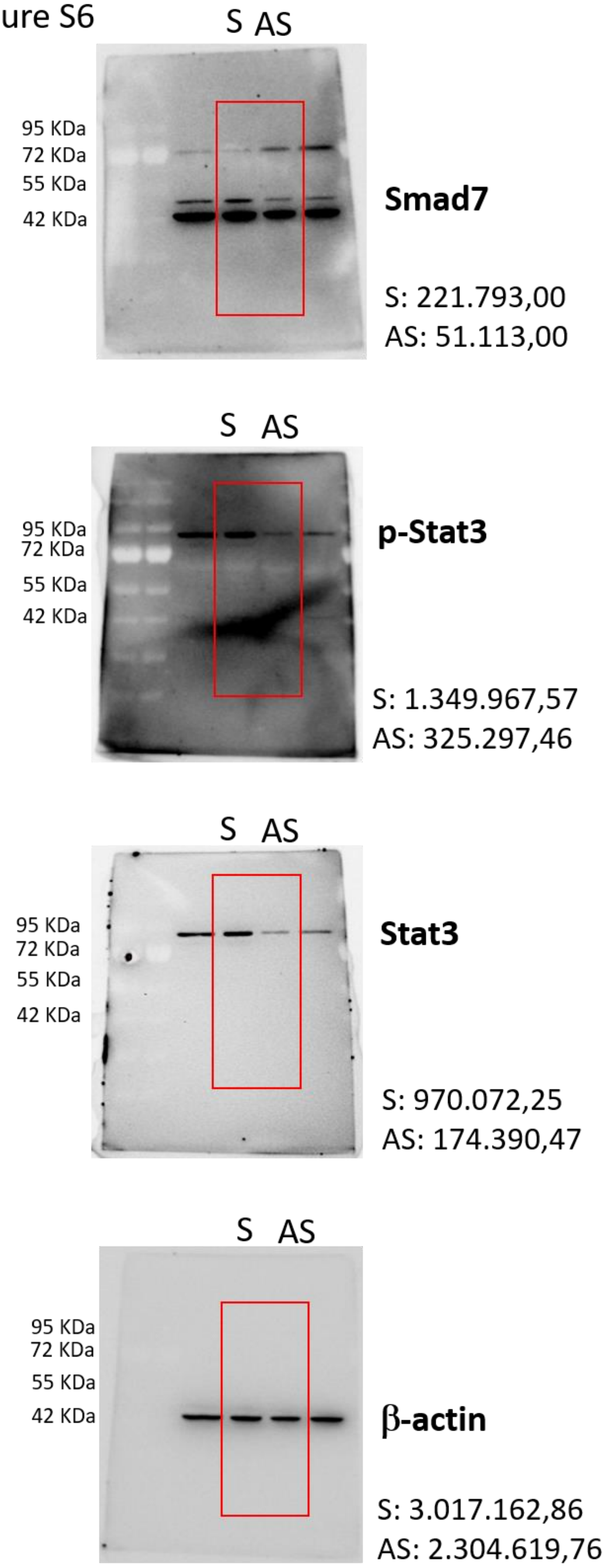
S AS
95 KDa
72 KDa
55 KDa
42 KDa
Smad7
S: 221.793,00
AS: 51.113,00
S AS
95 KDa
72 KDa
55 KDa
42 KDa
p-Stat3
S: 1.349.967,57
AS: 325.297,46
S AS
95 KDa
72 KDa
55 KDa
42 KDa
Stat3
S: 970.072,25
AS: 174.390,47
S AS
95 KDa
72 KDa
55 KDa
42 KDa
β-actin
S: 3.017.162,86
AS: 2.304.619,76

Figure S7

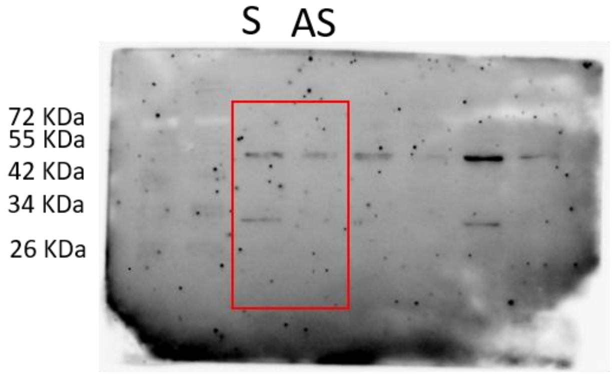

Smad7

S: 447.162,64
AS: 186.572,65

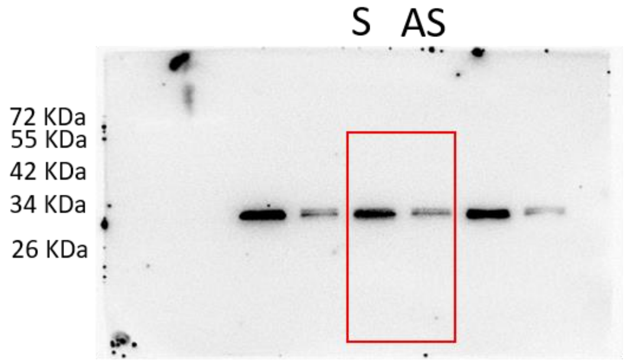

BCL XL

S: 2.288.875,08
AS: 727.602,00

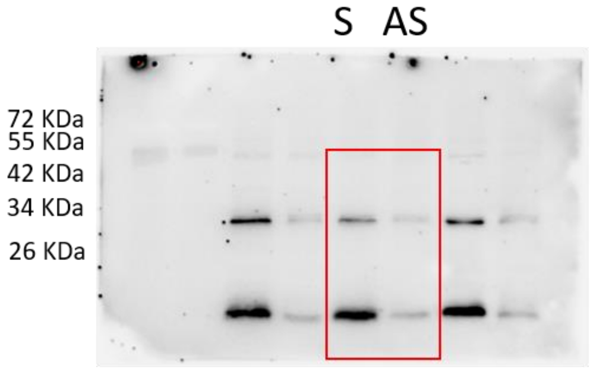

Survivn

S: 10.129.370,07
AS: 957.353,84

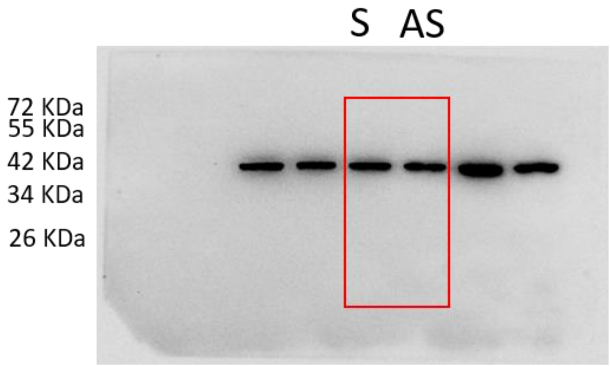

β-actin

S: 2.801.407,62
AS: 2.491.064,40
